# Evaluation of short-term epigenetic age fluctuation

**DOI:** 10.1186/s13148-022-01293-9

**Published:** 2022-06-09

**Authors:** Shohei Komaki, Hideki Ohmomo, Tsuyoshi Hachiya, Yoichi Sutoh, Kanako Ono, Ryohei Furukawa, So Umekage, Yayoi Otsuka-Yamasaki, Shiori Minabe, Akira Takashima, Kozo Tanno, Makoto Sasaki, Atsushi Shimizu

**Affiliations:** 1 Division of Biomedical Information Analysis, Iwate Tohoku Medical Megabank Organization, Disaster Reconstruction Center, Iwate Medical University, 1-1-1, Idaidori, Yahaba, Iwate 028-3694 Japan; 2 Division of Biomedical Information Analysis, Institute for Biomedical Sciences, Iwate Medical University, 1-1-1 Idaidori, Yahaba, Shiwa, Iwate 028-3694 Japan; 3 Department of Biology, Research and Education Center for Natural Sciences, Keio University, 4-1-1 Hiyoshi, Kohoku-ku, Yokohama, Kanagawa 223-8521 Japan; 4 Division of Clinical Research and Epidemiology, Iwate Tohoku Medical Megabank Organization, Iwate Medical University, Iwate, Japan; 5 Department of Hygiene and Preventive Medicine, Iwate Medical University, 1-1-1 Idaidori, Yahaba, Shiwa, Iwate 028-3694 Japan; 6 Iwate Tohoku Medical Megabank Organization, Iwate Medical University, Iwate, Japan; 7 Division of Ultrahigh Field MRI, Institute for Biomedical Sciences, Iwate Medical University, 1-1-1 Idaidori, Yahaba, Shiwa, Iwate 028-3694 Japan

**Keywords:** Blood DNA methylation, Epigenetic clock, Healthy individuals, Normal condition

## Abstract

Considerable effort has been spent on lowering and maintaining the epigenetic age. However, the extent to which epigenetic age fluctuates under normal conditions is poorly understood. Therefore, we analyzed methylation data from monocytes and peripheral blood mononuclear cells collected from two Japanese men. The ranges of the Pan-tissue, Skin and blood, and DNAm PhenoAge epigenetic age during 3 months were ≥ 5.62, ≥ 3.04, and ≥ 8.23 years, and the maximum daily changes were 5.21, 3.20, and 6.53 years, respectively. These fluctuations were not suppressed by correcting for cell-type composition. Although the underlying biological mechanism remains unclear, there was a nonnegligible degree of age fluctuation which should inform personalized clinical applications.

**Supplementary Information:**

The online version contains supplementary material available at 10.1186/s13148-022-01293-9.

## Introduction

DNA methylation has been utilized as a biomarker for age or age-related traits. Various models (clocks) currently exist, some of which have been developed to estimate chronological age and others to evaluate biological age. Therefore, the clocks can be used not only for determining age, but also for evaluating the acceleration of the aging rate by comparing epigenetic age with chronological age [1]. The effects of interventions such as exercise, lifestyle modification, and drug treatment on epigenetic age rejuvenation are of particular interest.

The effects of intervention are generally evaluated through observational studies, such as case–control comparisons and before and after intervention comparisons. However, little attention has been paid to longitudinal epigenetic age changes, which can occur without interventions. It is essential to determine the level of background fluctuation of epigenetic age under normal conditions to evaluate the effects of lifestyle interventions; if the estimated age changes markedly, even without intervention, it is more important to distinguish between the true effects of intervention and background fluctuations, especially in the personalized clinical scenario.

Herein, we evaluated short-term epigenetic age fluctuations using blood DNA methylation datasets collected at multiple time-points, and addressed the biological consequences of those fluctuations.

## Materials and methods

We analyzed the DNA methylation datasets obtained by Furukawa et al. [2]; they collected blood samples from two apparently healthy Japanese men (both in their 30 s) 24 times over 3 months, extracted peripheral blood mononuclear cells (PBMCs) and monocytes, and measured DNA methylation levels using Illumina Infinium® HumanMethylation450 BeadChip arrays (HM450k) (2 individuals × 2 sample types × 24 time-points = 96 samples) (Additional file 1: Methods 1). To prevent the identification of the two men, their chronological ages were not obtained. Their medication status and clinical conditions were also not collected, but they underwent blood tests on every blood collection day (Additional file 1: Table S1). No interventions were implemented except for overnight fasting and 24-h alcohol intake restriction prior to blood sampling. Thus, no intentional lifestyle change occurred during the sampling period. The samples were not randomly loaded on the microarrays but were ordered by collection date. The obtained DNA methylation profiles were normalized using a method proposed by Horvath [3] (Additional file 1: Methods 1).

We considered three clocks for the epigenetic age calculations: the Pan-tissue clock [3], the Skin and blood clock [4], and the DNAm PhenoAge clock [5]. The Pan-tissue and Skin & blood clocks were trained using chronological age, whereas the DNAm PhenoAge clock was trained using biological age. Thus, for achieving a more accurate biological age evaluation, the DNAm PhenoAge clock is preferred. However, many epigenetic age studies usually refer to and compare multiple clocks to evaluate the effect of external factors of interest on the epigenetic age acceleration. The prediction accuracies of the Pan-tissue and DNAm PhenoAge clocks with regard to monocytes and PBMCs were corroborated in the researchers' original articles. The accuracy of the Skin & blood clock was not evaluated in the original article.

To evaluate epigenetic age fluctuation, we calculated the range and coefficient of variation (CV) of the estimated age of each sample type (PBMC or monocyte) from each person (Person A and Person B). We also calculated the epigenetic age difference between each consecutive pair of blood-collection points, and estimated possible maximum epigenetic age changes over 1 day. Because the normalization method can affect the prediction accuracy and resultant degree of age fluctuation, we also evaluated epigenetic age fluctuations based on raw (unnormalized) and beta-mixture quantile-normalized datasets (Additional file 1: Methods 1), as well as the Horvath-normalized dataset mentioned above. To determine whether the fluctuation of epigenetic age resulted from biological consequences (in vivo) rather than experimental noise, we characterized the clock CpGs with higher and lower longitudinal methylation changes; in each clock, we extracted clock CpGs with greater (top 25%; variable CpGs) and smaller (bottom 25%; stable CpGs) standard deviations (SDs), and obtained the CpG and genic annotations of each CpG group as done in the previous study [6]. We then used the chi-squared test to evaluate differences in annotation proportions. The significant threshold was adjusted using the Bonferroni correction (3 clocks × 2 sample types = 6 tests; significant threshold = 0.05/6 = 0.008).

We also evaluated the contribution of the blood cell type proportion (Additional file 1: Fig. S1) to the epigenetic age fluctuation. We corrected the epigenetic ages for the cell type proportion, and compared the degrees of longitudinal fluctuation of corrected- and uncorrected-epigenetic ages (Additional file 1: Methods 2). The corrected- and uncorrected-epigenetic ages were further compared to the blood test results and basal body temperature (Additional file 1: Methods 3 and Additional file 1: Table S1).

We further investigated the mechanisms underlying the short-term epigenetic age fluctuations. We defined the contribution to the epigenetic age fluctuation as the product of the coefficient used in each clock and the SD (longitudinal change) of each clock CpG and extracted the CpGs that made the highest (top 25%) and lowest (bottom 25%) contributions. According to the procedure mentioned above, we obtained CpG and genic annotations pertaining to the CpGs, and tested the proportion difference of annotations. We also performed enrichment analyses of the two CpG groups that made the highest and lowest contributions. The procedure of the analyses is described in Additional file 1: Methods 4.

In the HM450k array, two different types of assays were used: Infinium I uses two probe types per target CpG site, whereas Infinium II uses a single type. This difference is known to affect replicability in DNA methylation measurements. Therefore, we compared the contributions made to the epigenetic fluctuations by clock CpGs with different assay types using Wilcoxon-Mann–Whitney tests.

All statistical analyses in this study were carried out using R version 4.0.5. Details of data processing are described in Additional file 1: Methods.

## Results and discussion

**Fig. 1:**
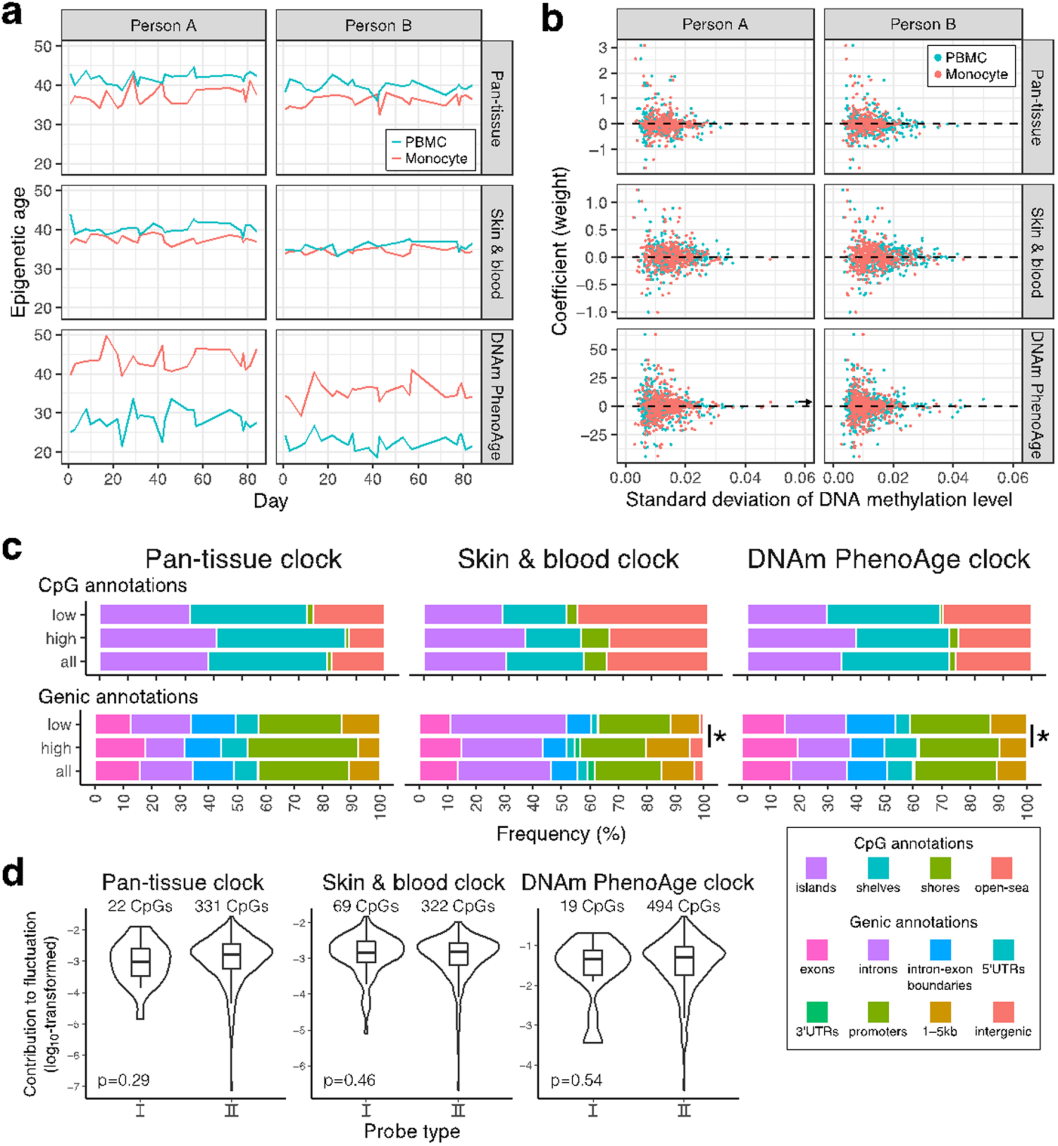
Epigenetic age fluctuation and underlying characteristics based on Horvath-normalized datasets. **a** Longitudinal change of epigenetic ages in peripheral blood mononuclear cells (PBMCs) and monocytes in the two individuals (Person A and Person B). The ages were calculated based on three clocks. **b** Relationships between longitudinal DNA methylation change (standard deviation: SD) and the coefficient assigned to the clock CpGs. The dashed line indicates coefficient = 0. The single DNAm PhenoAge clock CpG exhibited a greater SD in the PBMCs of Person A (> 0.15), as is indicated by an arrow. **c** Proportions of CpG and genic annotations of all clock CpGs (all), CpGs with a higher contribution to epigenetic age fluctuation (top 25% CpGs; high), and CpGs with a lower contribution (bottom 25% CpGs; low). Asterisks indicate a significant difference in annotation proportion. **d** Contribution of CpGs with type I and II probes to epigenetic age fluctuation (SD × coefficient)

According to the Pan-tissue clock with Horvath-normalized datasets, both Person A and Person B had an epigenetic age of approximately 35–45 years (Fig. 1a), although we did not obtain their chronological ages. The PBMCs indicated significantly older epigenetic ages than the monocytes (paired t-test *p*-values = 1.902 × 10^–9^ and 3.916 × 10^–9^ for Persons A and B, respectively). This sample type difference seems to corroborate the results of a previous study that compared the epigenetic ages calculated from whole blood and monocytes, and reported that the Pan-tissue age derived from the monocytes was younger than that derived from the whole blood [7]. The range of the short-term change of epigenetic age derived from monocytes in Person B was smallest (5.624) and largest in Person A (8.355; Table 1). The greatest epigenetic age change occurred in monocyte of Person B between Days 42 and 43 (5.215 years; Table 1). The relationship of the magnitude of fluctuation between monocyte and PBMC samples was not consistent between the two individuals (Table 1).

**Table 1:** Epigenetic age fluctuation of each sample according to the three clocks

	Monocytes		PBMCs	
	Person A	Person B	Person A	Person B
**Pan-tissue clock**				
Range	8.355	5.624	5.972	6.588
CV	0.060	0.042	0.034	0.037
Daily change	3.687	5.215	2.602	2.428
**Skin and blood clock**				
Range	3.738	3.039	6.076	4.393
CV	0.026	0.023	0.034	0.030
Daily change	1.862	2.305	3.197	2.672
**DNAm PhenoAge clock**				
Range	10.313	11.697	12.002	8.225
CV	0.058	0.069	0.105	0.085
Daily change	5.956	6.534	5.522	5.324

Results based on Horvath-normalized datasets are presented. Full results are provided in Additional file 1: Table S2.

*Range *the difference between the oldest and youngest epigenetic ages observed across 3 months*, PBCMs* peripheral blood mononuclear cells, *CV* coefficient of variance, *Daily change* the maximum epigenetic age change between two consecutive blood-collection days

The epigenetic age calculated using the Skin & blood clock was relatively longitudinally stable compared to the epigenetic ages calculated using the other two clocks (Fig. 1a and Table 1). The PBMCs of Person A had the largest range of epigenetic age change (6.076) and the greatest daily age change (3.197 between days 78 and 79) (Fig. 1a and Table 1). The difference in estimated age between the sample types (monocytes vs PBMCs) was smaller according to the Skin & blood clock than according to the other two clocks although there are still significant differences between the sample types (p-values = 5.876 × 10^–8^ and 8.674 × 10^–5^ in Persons A and B, respectively). For both Persons A and B, the magnitude of fluctuation was larger in PBMC samples (Table 1). According to the DNAm PhenoAge clock, the PBMCs of Person A and the monocytes of Person B indicated the greatest range within 80 days (12.002) and the greatest daily change (6.534), respectively (Fig. 1a and Table 1). Based on CVs, PBMC samples exhibited greater fluctuation; however, it was not consistent across indices (Table 1). Contrary to the Pan-tissue and Skin & blood clocks, according to the DNAm PhenoAge clock the PBMCs were approximately 20 years younger than the monocytes (*p*-values = 1.257 × 10^–15^ and 2.688 × 10^–17^, respectively). A comparison of DNAm PhenoAges derived from whole blood and monocytes also suggested that the DNAm PhenoAge of whole blood is younger than that of monocytes [7].

Roshandel et al. [7] also reported that the ages derived from whole blood decreased in the order: DNAm PhenoAge-, Skin & blood-, and Pan-tissue-estimated age. In contrast, when the ages were derived from monocytes, the Pan-tissue- and Skin & blood-estimated ages were compatible, and the DNAm PhenoAge was greatest. The sample type differences were similar in the PBMCs and monocytes in this study.

The epigenetic age was strongly influenced by the normalization strategy. In particular, unnormalized DNA methylation datasets produced remarkedly older Pan-tissue and Skin & blood ages and younger DNAm PhenoAges compared to normalized datasets (Additional file 1: Fig. S2). However, there was no marked difference in the degree of fluctuation between the normalization strategies (Additional file 1: Table S2).

The epigenetic ages corrected for cell type proportions showed slightly reduced ranges of age-change within 80 days compared to uncorrected epigenetic ages; however, no statistically significant reduction of longitudinal variation was observed (Additional file 1: Table S3). Furthermore, corrected epigenetic age showed greater maximum daily changes. These results imply that while cell type proportion is regarded to affect epigenetic age, it does not explain the short-term fluctuation of the epigenetic age.

Our study implies that there is considerable short-term epigenetic age fluctuation. For example, growth hormone administration had a rejuvenating effect on epigenetic age in PBMCs over 12 months (Pan-tissue clock: −2.50 ± 0.40 years; DNAm PhenoAge clock: −3.73 ± 1.26 years) [8]. Several whole blood studies have also reported epigenetic age changes following interventions that are comparable to those observed within a person over 3 months. For example, a dietary intervention study revealed that the epigenetic age decreased by 2.70 years in women with a specific genotype [9]. More comprehensive interventions, including those related to diet, sleep, exercise, and the administration of supplements for 8 weeks, reduced the epigenetic age by 1.96 years on average [10]. These results were obtained from between-group comparisons and were statistically significant; they therefore reflect the effects of the interventions. However, our study highlights that the same magnitude of change can occur within months or days without intervention. Therefore, short-term epigenetic age fluctuations have a nonnegligible effect, which reduces the ability to determine the effect of intervention on epigenetic ages.

The significant association with phenotype was only observed between Skin & blood clock age in PBMCs (corrected for cell type proportion) and γ-GTP levels (*p*-value = 7.11 × 10^–6^; Additional file 1: Tables S4–S6). However, whether the epigenetic age fluctuation is truly related to the daily fluctuation of γ-GTP levels within the normal range must be carefully considered. Since our results were obtained from only two persons, we avoid making conclusions.

In all three clocks, smaller coefficients were assigned for clock CpGs with large SDs in DNA methylation levels (Fig. 1b). Although these clocks were developed without explicitly considering short-term changes within individuals, they were ultimately designed to minimize the impact of short-term fluctuations in DNA methylation levels. The clock CpGs with greater and smaller longitudinal methylation level changes (SD) exhibited markedly different proportions of CpGs and genic annotations (*p*-values = 9.4 × 10^–4^–2.3 × 10^–9^; Additional file 1: Fig. S3), confirming that biological, rather than technical, factors are responsible for the observed changes in DNA methylation levels [6]. However, regarding contributions to the change in epigenetic age (coefficient × SD), there was no significant difference in the annotation proportions between the CpGs with higher and lower contributions, except for in two comparisons (genic annotations for the Pan-tissue and DNAm PhenoAge clock CpGs) (Fig. 1c). Enrichment analyses of the CpGs that made higher and lower contributions to the epigenetic age fluctuations also revealed no significantly enriched terms or pathways in the present study.CpGs captured by the Infinium II assay are known to be unstable compared to those captured by the Infinium I assay. However, the type II assay CpGs did not make greater contributions to the epigenetic age fluctuations in all three clocks (Fig. 1d). Therefore, the effects of assay type difference on epigenetic age fluctuation in each clock were apparently limited. Our study revealed that epigenetic ages can vary considerably. Although the observed fluctuations may occur in vivo, no unified biological consequence was identified. This indicates that complicated biological factors contribute to the variation in age estimates. Indeed, it has been suggested that a number of factors (e.g., smoking, mental stress, cancer, and physical activity) influence epigenetic age acceleration [1], indicating that the underlying mechanisms are intermingled. Considering such potential background, the lack of medical, medication, or lifestyle information on the subjects is a limitation of this study. Thus, the factors contributing to the fluctuation are unclear in this study. However, the important point remains that epigenetic age can change by > 3 years from day to day in apparently healthy individuals without intervention. Although the short-term epigenetic age fluctuations may be negligible in population-based studies, they have a significant influence on individual-level interventions and age assessments. The importance and usefulness of epigenetic age are not undermined by the present study. Our study verifies the importance of considering the possible fluctuations within individuals in the personalized clinical use of epigenetic age.

This study was based on only two Japanese males, both in their 30 s, which may have resulted in bias. Further studies on diverse populations are required to understand the implications of epigenetic age fluctuation, i.e., whether the fluctuation is undesirable noise in clinical applications or has potential as a high-sensitive biomarker reflecting clinical conditions at the time of blood collection.

## Supplementary Information

**Additional file 1: Supplementary information**. **Methods 1.** DNA methylation measurements; **Methods 2.** Contribution of cell-type proportion to the epigenetic age; **Methods 3.** Association between epigenetic age and blood test results; **Methods 4.** Annotation of CpGs and enrichment analyses. **Figure S1.** Estimated cell proportions in each sample; **Figure S2**. Epigenetic ages under each normalization method; **Figure S3.** Proportions of CpG and genic annotations. **Table S1.** Blood test items analyzed in this study; **Table S2.** Epigenetic age fluctuation of each sample under three epigenetic clocks and three normalization methods; **Table S3.** Epigenetic age fluctuation of each PBMC sample after correcting for cell-type proportion; **Table S4.** Associations between Pan-tissue clock epigenetic age and blood test results; **Table S5.** Associations between Skin & blood clock epigenetic age and blood test results; **Table S6.** Associations between DNAm PhenoAge and blood test results.

## Data Availability

Individual-level data cannot be made publicly available owing to informed consent. R codes used in this study are available on GitHub (https://github.com/ShoheiKomaki/ClinicalEpigenetics_2022).

## Abbreviations

CpG: Cytosine-phosphate-guanine
CV: Coefficient of variation
HM450k: Illumina Infinium® HumanMethylation450 BeadChip array
PBMC: Peripheral blood mononuclear cell
SD: Standard deviation
