# Supplementary material for: Evaluation of short-term epigenetic age fluctuation
Source: Clin Epigenetics. 2022 Jun 9;14:76. doi: 10.1186/s13148-022-01293-9 (PMC9185970; doi:10.1186/s13148-022-01293-9)
Supplement: Supplementary file 1 — Additional file 1: Supplementary information. Methods 1. DNA methylation measurements; Methods 2. Contribution of cell-type proportion to the epigenetic age; Methods 3. Association between epigenetic age and blood test results; Methods 4. Annotation of CpGs and enrichment analyses. Figure S1. Estimated cell proportions in each sample; Figure S2. Epigenetic ages under each normalization method; Figure S3. Proportions of CpG and genic annotations. Table S1. Blood test items analyzed in this study; Table S2. Epigenetic age fluctuation of each sample under three epigenetic clocks and three normalization methods; Table S3. Epigenetic age fluctuation of each PBMC sample after correcting for cell-type proportion; Table S4. Associations between Pan-tissue clock epigenetic age and blood test results; Table S5. Associations between Skin & blood clock epigenetic age and blood test results; Table S6. Associations between DNAm PhenoAge and blood test results. [file 13148_2022_1293_MOESM1_ESM.doc]

**SUPPLEMENTARY METHODS**

1. DNA methylation measurements

DNA methylation profiles were obtained in the previous study (1). From blood samples collected 24 times over 84 days from two Japanese men, PBMCs and monocytes were extracted. From each PBMC and monocyte sample, DNA was extracted using AllPrep®DNA/RNA Micro Kits (QIAGEN, Venlo, Netherlands), was subjected to the bisulfite conversion using EZ DNA Methylation Kit (Zymo Research, CA, USA), and hybridized into the Illumina Infinium® HumanMethylation450 BeadChip arrays (Illumina, CA, USA). The raw intensity data (IDAT) files were processed (color balance adjustment, background noise correction, quantile normalization) using the Bioconductor package "lumi" v2.14.2 (2) in R v3.0.2, and beta values (methylated probe vs. total signal intensities ratio) were obtained for each CpG.

Beta values were then normalized by two methods: beta-mixture quantile-normalization (BMIQ) and the method described by Horvath (3). BMIQ-normalization was carried out using a source code (BMIQ_1.3.R) of the function *BMIQ()* (4) in the R package "wateRmelon." Horvath-normalization was carried out using an R code (NORMALIZATION.R) downloaded from Horvath's website (https://horvath.genetics.ucla.edu/html/dnamage/) on November 3, 2021.

2. Contribution of cell-type proportion to the epigenetic age

The cell type proportion of PBMC samples, including B cell, CD4T, CD8T, monocyte, neutrophil, NK cell, was estimated from DNA methylation profiles in a previous study (1) using the *estimateCellCounts()* function in the R package "minfi" (5). We regressed the epigenetic ages on the cell type proportion using a multiple-liner regression model and obtained residuals (i.e., epigenetic age after subtracting the portion explained by cell type proportion). We defined this value as the epigenetic age corrected for cell-type proportion and compared it to the uncorrected epigenetic age. Using corrected epigenetic ages, as well as the uncorrected ages, we calculated the range (maximum minus minimum epigenetic ages observed within 80 days), standard deviation (SD), and the maximum epigenetic age change over one day. Since the coefficient of variation (CV) that was used to evaluate the fluctuation of uncorrected epigenetic age (see main text) can only be applied to positive values, CV was not considered for the corrected epigenetic age. SDs of corrected and uncorrected epigenetic ages were further compared via the F-test.

3. Association between epigenetic age and blood test results

On every blood collection day, two subjects had blood tests consisting of 12 items and measurements of basal body temperature (Table S1). Associations of the 13 variables and epigenetic ages (raw epigenetic ages of PBMCs and monocytes, and cell type proportion-corrected epigenetic ages of PBMCs) were evaluated by simple linear regression analyses. Based on the obtained coefficients with standard errors, we further conducted meta-analyses using R codes provided by Dr. Matti Pirinen (https://www.mv.helsinki.fi/home/mjxpirin/GWAS_course/material/GWAS9.html, accessed on 6 April 2022).

4. Annotation of CpGs and enrichment analyses

The CpG annotations (CpG islands, shores, shelves, open seas) and genic annotations (exons, introns, intron-exon boundaries, promoters, 3' and 5' UTRs, 1–5kb upstream of TSS, intergenic regions) of each CpG were performed using the R package "annotatr" v1.18.1 (6). Based on the genes annotated for CpGs of each CpG category, enrichment analyses were performed using the *gometh()* function in the R package "missMethyl" v1.26.1 (7). Gene Ontology annotations (including biological process, cellular component, and molecular function) and Kyoto Encyclopedia of Genes and Genomes pathways were applied in the analyses. Terms and pathways with a false discovery rate-adjusted p-value < 0.05 were considered to be significantly enriched.

**SUPPLEMENTARY FIGURES**

**Figure S1. Estimated cell proportions in each sample.** The estimations were carried out in the previous study (1) using the *estimateCellCounts()* function of an R package "minfi."

**Figure S2. Epigenetic ages under each normalization method.** (a) Epigenetic age changes under each clock and normalization method. (b–d) Relationships between longitudinal DNA methylation change (standard deviation: SD) and the coefficient assigned to clock CpGs under each normalization method. The dashed line indicates coefficient=0. Single Levine 2018 clock CpG exhibited greater SD in PBMC of Person A (>0.15) which is indicated by a diamond.

**Figure S3. Proportions of CpG and genic annotations.** Proportions in all clock CpGs (all), CpGs with a smaller SD (stable), and CpGs with greater SD (variable) in the Horvath-normalized dataset are presented. P values resulting from tests for annotation proportion difference between stable and variable CpGs are also presented.

**SUPPLEMENTARY TABLES**

**Table S1. Blood test items analyzed in this study.**

| Item (unit) | Abbreviation | Person A | Person B |
| --- | --- | --- | --- |
| Body temperature (°C) | BT | 36.27 ± 0.22 | 36.2 ± 0.15 |
| Glutamic-oxaloacetic transaminase (U/L) | GOT | 16.71 ± 3.01 | 19.08 ± 1.35 |
| Glutamic-pyruvic transaminase (U/L) | GPT | 13.46 ± 3.48 | 18.29 ± 2.16 |
| γ-glutamyl transpeptidase (U/L) | γGTP | 15.08 ± 1.28 | 13.5 ± 1.69 |
| Creatinine (mg/dL) | CR | 0.68 ± 0.04 | 0.82 ± 0.04 |
| Uric acid (mg/dL) | UA | 6.32 ± 0.47 | 4.09 ± 0.31 |
| Blood urea nitrogen (mg/dL) | BUN | 9.85 ± 0.97 | 11.22 ± 1.84 |
| Total cholesterol (mg/dL) | TC | 189.5 ± 9.04 | 163.29 ± 9.16 |
| HDL cholesterol (mg/dL) | HDL | 47.63 ± 2.81 | 40.88 ± 2.25 |
| LDL cholesterol (mg/dL) | LDL | 119.92 ± 10.05 | 98.71 ± 8.17 |
| Triglyceride (mg/dL) | TG | 159.25 ± 36.64 | 136.5 ± 25.25 |
| Total IgE antibody (IU/mL) | IgE | 365.58 ± 33.49 | 81.08 ± 9.21 |
| High sensitive CRP (mg/L) | hsCRP | 0.1 ± 0.22 | 0.11 ± 0.32 |

Mean ± standard deviation is presented.

**Table S2. Epigenetic age fluctuation of each sample under three epigenetic clocks and three normalization methods.**

|  |  | Monocyte |  |  |  |  |  | PBMC |  |  |  |  |  |
| --- | --- | --- | --- | --- | --- | --- | --- | --- | --- | --- | --- | --- | --- |
|  |  | Person A |  |  | Person B |  |  | Person A |  |  | Person B |  |  |
|  |  | Raw | BMIQ | Horvath | Raw | BMIQ | Horvath | Raw | BMIQ | Horvath | Raw | BMIQ | Horvath |
| Pan-tissue clock | Range | 8.04 | 8.00 | 8.36 | 5.87 | 6.22 | 5.62 | 6.14 | 6.36 | 5.97 | 7.08 | 7.19 | 6.59 |
|  | SD | 2.11 | 2.04 | 2.22 | 1.58 | 1.57 | 1.51 | 1.48 | 1.59 | 1.44 | 1.56 | 1.60 | 1.48 |
|  | CV | 0.06 | 0.05 | 0.06 | 0.05 | 0.04 | 0.04 | 0.04 | 0.04 | 0.03 | 0.04 | 0.04 | 0.04 |
|  | Daily | 3.64 | 3.37 | 3.69 | 5.82 | 5.66 | 5.21 | 2.63 | 2.94 | 2.60 | 2.31 | 2.66 | 2.43 |
| Skin & blood clock | Range | 3.32 | 3.36 | 3.74 | 3.69 | 4.26 | 3.04 | 4.72 | 4.83 | 6.08 | 4.76 | 4.82 | 4.39 |
|  | SD | 1.01 | 0.96 | 0.98 | 0.95 | 1.02 | 0.79 | 1.20 | 1.22 | 1.38 | 1.28 | 1.31 | 1.08 |
|  | CV | 0.03 | 0.02 | 0.03 | 0.03 | 0.03 | 0.02 | 0.03 | 0.03 | 0.03 | 0.04 | 0.03 | 0.03 |
|  | Daily | 2.12 | 1.88 | 1.86 | 2.50 | 2.79 | 2.31 | 3.26 | 3.26 | 3.20 | 2.73 | 2.90 | 2.67 |
| DNAm PhenoAge  clock | Range | 10.48 | 7.92 | 10.31 | 13.03 | 10.50 | 11.70 | 12.18 | 12.80 | 12.00 | 8.87 | 9.01 | 8.23 |
|  | SD | 2.61 | 2.19 | 2.53 | 2.70 | 2.41 | 2.44 | 3.07 | 3.07 | 2.94 | 2.10 | 2.06 | 1.91 |
|  | CV | 0.06 | 0.05 | 0.06 | 0.08 | 0.07 | 0.07 | 0.11 | 0.12 | 0.10 | 0.10 | 0.11 | 0.08 |
|  | Daily | 5.65 | 5.51 | 5.96 | 7.18 | 6.00 | 6.53 | 5.90 | 5.20 | 5.52 | 6.12 | 6.04 | 5.32 |

Range: the difference between oldest and youngest epigenetic age observed across three months. SD: standard deviation. CV: coefficient of variance. Daily change: the maximum epigenetic age change between two consecutive blood-collection days.

**Table S3. Epigenetic age fluctuation of each PBMC sample after correcting for cell-type proportion.**

|  |  | Person A |  |  | Person B |  |  |
| --- | --- | --- | --- | --- | --- | --- | --- |
|  |  | Raw | BMIQ | Horvath | Raw | BMIQ | Horvath |
| Pan-tissue clock | Range | 5.46 | 5.87 | 5.33 | 7.02 | 7.07 | 6.52 |
|  | SD | 1.30 | 1.41 | 1.26 | 1.51 | 1.55 | 1.39 |
|  | (F-test p value*) | 0.53 | 0.55 | 0.53 | 0.87 | 0.87 | 0.77 |
|  | Daily | 3.66 | 3.56 | 3.48 | 3.66 | 3.56 | 3.48 |
| Skin & blood clock | Range | 4.03 | 4.16 | 5.97 | 4.71 | 4.75 | 4.14 |
|  | SD | 1.17 | 1.15 | 1.40 | 1.32 | 1.36 | 1.15 |
|  | (F-test p value*) | 0.92 | 0.79 | 0.95 | 0.87 | 0.88 | 0.76 |
|  | Daily | 3.61 | 3.71 | 5.49 | 3.61 | 3.71 | 5.49 |
| DNAm PhenoAge clock | Range | 9.04 | 8.81 | 8.71 | 7.38 | 7.51 | 6.67 |
|  | SD | 2.40 | 2.31 | 2.27 | 2.04 | 1.98 | 1.83 |
|  | (F-test p value*) | 0.25 | 0.18 | 0.22 | 0.89 | 0.85 | 0.84 |
|  | Daily | 7.42 | 7.72 | 7.38 | 7.42 | 7.72 | 7.38 |

*F-test was performed to compare the degree of longitudinal variations of raw epigenetic age (presented in Table S1) and cell-type-corrected epigenetic age. Raw p values are presented.

Range: the difference between oldest and youngest epigenetic age observed across three months. SD: standard deviation. CV: coefficient of variance. Daily change: the maximum epigenetic age change between two consecutive blood-collection days.

**Table S4. Associations between Pan-tissue clock epigenetic age and blood test results.**

|  |  | Person A |  |  | Person B |  |  | Meta-analysis | |  |
| --- | --- | --- | --- | --- | --- | --- | --- | --- | --- | --- |
| Epigenetic age | Variable | Coefficient | SE | P value | Coefficient | SE | P value | Coefficient | SE | P value |
| Monocyte | BT | 1.43 | 2.15 | 0.51 | 0.62 | 2.13 | 0.77 | 1.02 | 1.51 | 0.50 |
|  | GOT | 0.15 | 0.15 | 0.35 | -0.41 | 0.22 | 0.08 | -0.03 | 0.13 | 0.79 |
|  | GPT | 0.14 | 0.13 | 0.31 | -0.23 | 0.14 | 0.12 | -0.03 | 0.10 | 0.73 |
|  | γGTP | 0.15 | 0.37 | 0.68 | -0.02 | 0.19 | 0.93 | 0.02 | 0.17 | 0.91 |
|  | CR | -24.87 | 11.43 | 0.04 | 0.01 | 7.15 | 1.00 | -7.00 | 6.06 | 0.25 |
|  | UA | -0.49 | 1.01 | 0.63 | 0.34 | 1.02 | 0.74 | -0.08 | 0.72 | 0.91 |
|  | BUN | -0.62 | 0.47 | 0.20 | 0.02 | 0.17 | 0.92 | -0.06 | 0.16 | 0.72 |
|  | TC | 0.02 | 0.05 | 0.75 | -0.07 | 0.03 | 0.03 | -0.05 | 0.03 | 0.07 |
|  | HDL | 0.29 | 0.16 | 0.08 | -0.07 | 0.14 | 0.64 | 0.09 | 0.11 | 0.38 |
|  | LDL | 0.02 | 0.05 | 0.69 | -0.06 | 0.04 | 0.11 | -0.03 | 0.03 | 0.29 |
|  | TG | -0.01 | 0.01 | 0.33 | -0.02 | 0.01 | 0.11 | -0.02 | 0.01 | 0.06 |
|  | IgE | -0.01 | 0.01 | 0.66 | 0.01 | 0.03 | 0.77 | 0.00 | 0.01 | 0.76 |
|  | hsCRP | -0.64 | 2.15 | 0.77 | 0.74 | 0.98 | 0.46 | 0.50 | 0.89 | 0.57 |
| PBMC | BT | 0.68 | 1.40 | 0.63 | -4.83 | 1.83 | 0.02 | -1.36 | 1.11 | 0.22 |
|  | GOT | 0.11 | 0.10 | 0.28 | -0.11 | 0.23 | 0.65 | 0.08 | 0.09 | 0.40 |
|  | GPT | 0.11 | 0.09 | 0.21 | -0.03 | 0.15 | 0.82 | 0.07 | 0.07 | 0.32 |
|  | γGTP | 0.07 | 0.24 | 0.76 | 0.32 | 0.17 | 0.08 | 0.23 | 0.14 | 0.10 |
|  | CR | -8.36 | 7.97 | 0.31 | 5.81 | 6.93 | 0.41 | -0.29 | 5.23 | 0.96 |
|  | UA | 0.22 | 0.66 | 0.74 | 0.95 | 0.98 | 0.34 | 0.45 | 0.55 | 0.41 |
|  | BUN | 0.45 | 0.30 | 0.15 | -0.15 | 0.17 | 0.37 | -0.01 | 0.15 | 0.95 |
|  | TC | -0.04 | 0.03 | 0.28 | 0.00 | 0.03 | 0.92 | -0.02 | 0.02 | 0.38 |
|  | HDL | 0.09 | 0.11 | 0.41 | -0.26 | 0.13 | 0.06 | -0.05 | 0.08 | 0.54 |
|  | LDL | -0.02 | 0.03 | 0.57 | 0.02 | 0.04 | 0.70 | -0.01 | 0.02 | 0.83 |
|  | TG | -0.01 | 0.01 | 0.17 | 0.00 | 0.01 | 0.83 | -0.01 | 0.01 | 0.28 |
|  | IgE** | -0.02 | 0.01 | 6.19E-03 | 0.05 | 0.03 | 0.18 | -0.02 | 0.01 | 8.49E-03 |
|  | hsCRP | 1.86 | 1.34 | 0.18 | -0.99 | 0.95 | 0.31 | -0.03 | 0.78 | 0.97 |
| Cor.PBMC^1^ | BT | 0.81 | 1.22 | 0.52 | -3.74 | 1.81 | 0.05 | -0.62 | 1.01 | 0.54 |
|  | GOT | 0.09 | 0.09 | 0.29 | -0.19 | 0.22 | 0.38 | 0.05 | 0.08 | 0.51 |
|  | GPT | 0.10 | 0.07 | 0.21 | -0.03 | 0.14 | 0.84 | 0.07 | 0.07 | 0.29 |
|  | γGTP | 0.10 | 0.21 | 0.62 | 0.30 | 0.16 | 0.08 | 0.22 | 0.13 | 0.08 |
|  | CR | -9.87 | 6.83 | 0.16 | 5.06 | 6.53 | 0.45 | -2.08 | 4.72 | 0.66 |
|  | UA | -0.26 | 0.57 | 0.65 | 0.68 | 0.93 | 0.47 | 0.00 | 0.49 | 0.99 |
|  | BUN | 0.32 | 0.27 | 0.25 | -0.18 | 0.16 | 0.27 | -0.05 | 0.14 | 0.70 |
|  | TC | -0.04 | 0.03 | 0.21 | -0.01 | 0.03 | 0.83 | -0.02 | 0.02 | 0.27 |
|  | HDL | 0.06 | 0.09 | 0.52 | -0.16 | 0.13 | 0.21 | -0.02 | 0.08 | 0.80 |
|  | LDL | -0.02 | 0.03 | 0.38 | 0.01 | 0.04 | 0.82 | -0.01 | 0.02 | 0.55 |
|  | TG | -0.01 | 0.01 | 0.19 | 0.00 | 0.01 | 0.68 | -0.01 | 0.01 | 0.35 |
|  | IgE | -0.01 | 0.01 | 0.07 | 0.05 | 0.03 | 0.14 | -0.01 | 0.01 | 0.13 |
|  | hsCRP | 1.12 | 1.20 | 0.36 | -0.97 | 0.90 | 0.29 | -0.22 | 0.72 | 0.76 |

^1^Epigenetic age of PBMC samples corrected for blood cell counts

**Meta-analysis P value < 0.01

**Table S5. Associations between Skin & blood clock epigenetic age and blood test results.**

|  |  | Person A |  |  | Person B |  |  | Meta-analysis | |  |
| --- | --- | --- | --- | --- | --- | --- | --- | --- | --- | --- |
| Epigenetic age | Variable | Coefficient | SE | P value | Coefficient | SE | P value | Coefficient | SE | P value |
| Monocyte | BT | -0.38 | 0.96 | 0.70 | 1.57 | 1.08 | 0.16 | 0.48 | 0.71 | 0.50 |
|  | GOT | 0.02 | 0.07 | 0.72 | 0.10 | 0.12 | 0.45 | 0.04 | 0.06 | 0.49 |
|  | GPT | -0.05 | 0.06 | 0.42 | -0.03 | 0.08 | 0.67 | -0.04 | 0.05 | 0.36 |
|  | γGTP | 0.06 | 0.16 | 0.72 | -0.17 | 0.09 | 0.07 | -0.12 | 0.08 | 0.15 |
|  | CR | 2.00 | 5.54 | 0.72 | -4.50 | 3.65 | 0.23 | -2.53 | 3.05 | 0.41 |
|  | UA | 0.39 | 0.44 | 0.39 | -0.15 | 0.54 | 0.79 | 0.17 | 0.34 | 0.61 |
|  | BUN | 0.02 | 0.22 | 0.93 | 0.14 | 0.09 | 0.12 | 0.12 | 0.08 | 0.13 |
|  | TC | 0.04 | 0.02 | 0.12 | -0.01 | 0.02 | 0.69 | 0.01 | 0.01 | 0.46 |
|  | HDL | 0.01 | 0.07 | 0.93 | 0.03 | 0.07 | 0.70 | 0.02 | 0.05 | 0.73 |
|  | LDL | 0.03 | 0.02 | 0.10 | -0.02 | 0.02 | 0.33 | 0.01 | 0.01 | 0.57 |
|  | TG | 0.00 | 0.01 | 0.75 | 0.00 | 0.01 | 0.82 | 0.00 | 0.00 | 0.92 |
|  | IgE | 0.01 | 0.01 | 0.07 | -0.02 | 0.02 | 0.19 | 0.01 | 0.01 | 0.16 |
|  | hsCRP | -0.34 | 0.95 | 0.72 | 0.07 | 0.52 | 0.90 | -0.03 | 0.46 | 0.95 |
| PBMC | BT | 0.00 | 1.35 | 1.00 | 1.95 | 1.47 | 0.20 | 0.89 | 1.00 | 0.37 |
|  | GOT | 0.08 | 0.10 | 0.43 | -0.25 | 0.16 | 0.14 | -0.01 | 0.08 | 0.94 |
|  | GPT | 0.00 | 0.08 | 0.97 | -0.20 | 0.10 | 0.05 | -0.09 | 0.06 | 0.18 |
|  | γGTP*** | -0.35 | 0.22 | 0.12 | -0.38 | 0.11 | 1.97E-03 | -0.38 | 0.10 | 1.15E-04 |
|  | CR | 9.52 | 7.58 | 0.22 | -6.21 | 4.95 | 0.22 | -1.50 | 4.15 | 0.72 |
|  | UA | 0.56 | 0.62 | 0.37 | 0.05 | 0.73 | 0.95 | 0.35 | 0.47 | 0.46 |
|  | BUN | 0.03 | 0.30 | 0.92 | 0.17 | 0.12 | 0.18 | 0.15 | 0.11 | 0.18 |
|  | TC | -0.03 | 0.03 | 0.42 | -0.04 | 0.02 | 0.11 | -0.04 | 0.02 | 0.06 |
|  | HDL | 0.08 | 0.10 | 0.48 | 0.13 | 0.10 | 0.19 | 0.10 | 0.07 | 0.14 |
|  | LDL | -0.01 | 0.03 | 0.82 | -0.05 | 0.03 | 0.08 | -0.03 | 0.02 | 0.12 |
|  | TG* | -0.02 | 0.01 | 0.02 | 0.00 | 0.01 | 0.91 | -0.01 | 0.01 | 0.05 |
|  | IgE** | -0.01 | 0.01 | 0.09 | -0.06 | 0.02 | 0.02 | -0.02 | 0.01 | 9.59E-03 |
|  | hsCRP | -0.11 | 1.34 | 0.93 | -0.39 | 0.71 | 0.59 | -0.33 | 0.63 | 0.60 |
| Cor.PBMC^1^ | BT | 0.20 | 1.37 | 0.88 | 1.82 | 1.59 | 0.26 | 0.90 | 1.04 | 0.39 |
|  | GOT | 0.05 | 0.10 | 0.60 | -0.25 | 0.17 | 0.17 | -0.02 | 0.09 | 0.82 |
|  | GPT | -0.02 | 0.09 | 0.78 | -0.24 | 0.10 | 0.03 | -0.11 | 0.07 | 0.09 |
|  | γGTP**** | -0.44 | 0.21 | 0.05 | -0.44 | 0.11 | 6.15E-04 | -0.44 | 0.10 | 7.11E-06 |
|  | CR | 9.47 | 7.69 | 0.23 | -11.59 | 4.88 | 0.03 | -5.55 | 4.12 | 0.18 |
|  | UA | 0.12 | 0.64 | 0.85 | -0.29 | 0.78 | 0.72 | -0.04 | 0.49 | 0.93 |
|  | BUN | -0.05 | 0.31 | 0.87 | 0.14 | 0.13 | 0.29 | 0.11 | 0.12 | 0.35 |
|  | TC | -0.03 | 0.03 | 0.30 | -0.02 | 0.03 | 0.37 | -0.03 | 0.02 | 0.17 |
|  | HDL | 0.02 | 0.11 | 0.83 | 0.20 | 0.10 | 0.06 | 0.12 | 0.07 | 0.11 |
|  | LDL | -0.01 | 0.03 | 0.62 | -0.04 | 0.03 | 0.23 | -0.03 | 0.02 | 0.22 |
|  | TG | -0.02 | 0.01 | 0.03 | 0.00 | 0.01 | 0.96 | -0.01 | 0.01 | 0.08 |
|  | IgE* | -0.01 | 0.01 | 0.31 | -0.07 | 0.02 | 3.22E-03 | -0.02 | 0.01 | 0.03 |
|  | hsCRP | -0.69 | 1.35 | 0.61 | -0.86 | 0.74 | 0.25 | -0.83 | 0.65 | 0.20 |

^1^Epigenetic age corrected for blood cell counts

*Meta-analysis P value < 0.05, **P < 0.01, ***P < 0.001, ****P<4.3×10^-4^ (Bonferroni-corrected significant threshold)

**Table S6. Associations between DNAm PhenoAge and blood test results.**

|  |  | Person A |  |  | Person B |  |  | Meta-analysis | |  |
| --- | --- | --- | --- | --- | --- | --- | --- | --- | --- | --- |
| Epigenetic age | Variable | Coefficient | SE | P value | Coefficient | SE | P value | Coefficient | SE | P value |
| Monocyte | BT | -3.75 | 2.35 | 0.12 | 6.33 | 3.19 | 0.06 | -0.20 | 1.89 | 0.91 |
|  | GOT | 0.02 | 0.18 | 0.91 | 0.01 | 0.39 | 0.97 | 0.02 | 0.16 | 0.91 |
|  | GPT | 0.09 | 0.15 | 0.56 | -0.33 | 0.23 | 0.17 | -0.04 | 0.13 | 0.76 |
|  | γGTP | 0.54 | 0.40 | 0.19 | -0.12 | 0.31 | 0.70 | 0.12 | 0.24 | 0.61 |
|  | CR | -9.30 | 14.24 | 0.52 | 10.99 | 11.37 | 0.34 | 3.09 | 8.88 | 0.73 |
|  | UA | -0.53 | 1.15 | 0.65 | 2.30 | 1.58 | 0.16 | 0.45 | 0.93 | 0.63 |
|  | BUN | -0.65 | 0.54 | 0.24 | 0.33 | 0.27 | 0.25 | 0.12 | 0.24 | 0.61 |
|  | TC | 0.06 | 0.06 | 0.29 | -0.11 | 0.05 | 0.04 | -0.03 | 0.04 | 0.37 |
|  | HDL | 0.08 | 0.19 | 0.68 | -0.02 | 0.23 | 0.95 | 0.04 | 0.15 | 0.78 |
|  | LDL | 0.06 | 0.05 | 0.22 | -0.11 | 0.06 | 0.08 | -0.01 | 0.04 | 0.77 |
|  | TG* | -0.02 | 0.01 | 0.16 | -0.03 | 0.02 | 0.14 | -0.02 | 0.01 | 0.04 |
|  | IgE | 0.00 | 0.02 | 0.76 | -0.01 | 0.06 | 0.84 | 0.00 | 0.02 | 0.81 |
|  | hsCRP* | 0.55 | 2.45 | 0.83 | 3.61 | 1.42 | 0.02 | 2.84 | 1.23 | 0.02 |
| PBMC | BT | -0.10 | 2.88 | 0.97 | 0.52 | 2.70 | 0.85 | 0.23 | 1.97 | 0.91 |
|  | GOT | 0.15 | 0.21 | 0.47 | 0.29 | 0.30 | 0.34 | 0.20 | 0.17 | 0.24 |
|  | GPT | -0.02 | 0.18 | 0.93 | 0.11 | 0.19 | 0.55 | 0.05 | 0.13 | 0.72 |
|  | γGTP* | -0.05 | 0.49 | 0.91 | 0.51 | 0.21 | 0.03 | 0.42 | 0.20 | 0.03 |
|  | CR | 2.22 | 16.65 | 0.90 | 7.33 | 8.93 | 0.42 | 6.19 | 7.87 | 0.43 |
|  | UA | 1.25 | 1.31 | 0.35 | 1.40 | 1.26 | 0.28 | 1.33 | 0.91 | 0.14 |
|  | BUN | 0.04 | 0.65 | 0.96 | -0.13 | 0.22 | 0.55 | -0.12 | 0.21 | 0.58 |
|  | TC | 0.00 | 0.07 | 0.97 | -0.02 | 0.04 | 0.67 | -0.01 | 0.04 | 0.70 |
|  | HDL | -0.27 | 0.21 | 0.22 | 0.02 | 0.18 | 0.91 | -0.10 | 0.14 | 0.47 |
|  | LDL | 0.01 | 0.06 | 0.93 | -0.03 | 0.05 | 0.51 | -0.02 | 0.04 | 0.64 |
|  | TG | -0.01 | 0.02 | 0.57 | 0.00 | 0.02 | 0.80 | -0.01 | 0.01 | 0.56 |
|  | IgE | -0.03 | 0.02 | 0.08 | 0.07 | 0.04 | 0.08 | -0.02 | 0.02 | 0.32 |
|  | hsCRP** | 2.74 | 2.78 | 0.34 | 2.90 | 1.10 | 0.01 | 2.88 | 1.02 | 4.77E-03 |
| Cor.PBMC^1^ | BT | -0.01 | 2.22 | 1.00 | -0.06 | 2.59 | 0.98 | -0.03 | 1.69 | 0.99 |
|  | GOT | 0.13 | 0.16 | 0.40 | 0.20 | 0.29 | 0.50 | 0.15 | 0.14 | 0.28 |
|  | GPT | 0.05 | 0.14 | 0.74 | 0.02 | 0.18 | 0.90 | 0.04 | 0.11 | 0.73 |
|  | γGTP | -0.03 | 0.38 | 0.93 | 0.24 | 0.22 | 0.29 | 0.17 | 0.19 | 0.37 |
|  | CR | -4.63 | 12.82 | 0.72 | -1.94 | 8.67 | 0.82 | -2.79 | 7.18 | 0.70 |
|  | UA | -0.05 | 1.04 | 0.96 | 0.57 | 1.23 | 0.65 | 0.20 | 0.79 | 0.80 |
|  | BUN | 0.39 | 0.49 | 0.44 | -0.12 | 0.21 | 0.59 | -0.04 | 0.19 | 0.84 |
|  | TC | -0.06 | 0.05 | 0.24 | 0.01 | 0.04 | 0.76 | -0.02 | 0.03 | 0.59 |
|  | HDL | -0.27 | 0.16 | 0.12 | 0.10 | 0.17 | 0.56 | -0.09 | 0.12 | 0.43 |
|  | LDL | -0.05 | 0.05 | 0.32 | 0.01 | 0.05 | 0.82 | -0.02 | 0.03 | 0.57 |
|  | TG | 0.00 | 0.01 | 0.97 | 0.00 | 0.02 | 0.80 | 0.00 | 0.01 | 0.85 |
|  | IgE | -0.02 | 0.01 | 0.25 | 0.02 | 0.04 | 0.71 | -0.01 | 0.01 | 0.32 |
|  | hsCRP | -1.17 | 2.18 | 0.60 | 1.18 | 1.18 | 0.33 | 0.65 | 1.04 | 0.53 |

^1^Epigenetic age corrected for blood cell counts

*Meta-analysis P value < 0.05, **P < 0.01

**REFERENCES**

1. Furukawa R, Hachiya T, Ohmomo H, Shiwa Y, Ono K, Suzuki S, et al. Intraindividual dynamics of transcriptome and genome-wide stability of DNA methylation. Sci Rep [Internet]. 2016;6(1):26424. Available from: http://www.nature.com/articles/srep26424

2. Du P, Kibbe WA, Lin SM. lumi: A pipeline for processing Illumina microarray. Bioinformatics. 2008;24(13):1547–8.

3. Horvath S. DNA methylation age of human tissues and cell types. Genome Biol [Internet]. 2013;14(10):R115. Available from: http://genomebiology.biomedcentral.com/articles/10.1186/gb-2013-14-10-r115

4. Teschendorff AE, Marabita F, Lechner M, Bartlett T, Tegner J, Gomez-Cabrero D, et al. A beta-mixture quantile normalization method for correcting probe design bias in Illumina Infinium 450 k DNA methylation data. Bioinformatics. 2013;29(2):189–96.

5. Aryee MJ, Jaffe AE, Corrada-Bravo H, Ladd-Acosta C, Feinberg AP, Hansen KD, et al. Minfi: A flexible and comprehensive Bioconductor package for the analysis of Infinium DNA methylation microarrays. Bioinformatics. 2014;30(10):1363–9.

6. Cavalcante RG, Sartor MA. Annotatr: Genomic regions in context. Bioinformatics. 2017;33(15):2381–3.

7. Phipson B, Maksimovic J, Oshlack A. MissMethyl: An R package for analyzing data from Illumina’s HumanMethylation450 platform. Bioinformatics. 2016;32(2):286–8.
